# Thyroid hormone increases oxygen metabolism causing intrarenal tissue hypoxia; a pathway to kidney disease

**DOI:** 10.1371/journal.pone.0264524

**Published:** 2022-03-03

**Authors:** Ebba Sivertsson, Malou Friederich-Persson, Patrik Persson, Masaomi Nangaku, Peter Hansell, Fredrik Palm

**Affiliations:** 1 Department of Medical Cell Biology, Uppsala University, Uppsala, Sweden; 2 Department of Medicine, University of Tokyo, Tokyo, Japan; University Medical Center Utrecht, NETHERLANDS

## Abstract

The proposed mechanisms for the development of nephropathy are many, complex and often overlapping. Although recent literature strongly supports a role of kidney hypoxia as an independent pathway to nephropathy, the evidence remains inconclusive since the role of hypoxia is difficult to differentiate from confounding factors such as hyperglycemia, hypertension and oxidative stress. By increasing kidney oxygen consumption using triiodothyronine (T_3_) and, thus, avoiding these confounding factors, the aim of the present study was to investigate renal hypoxia *per se* as a causal pathway for the development of nephropathy. Healthy Sprague-Dawley rats were treated with T_3_ (10 μg/kg/day) and the angiotensin II AT_1_-receptor antagonist candesartan (1 mg/kg in drinking water) to eliminate effects of T_3_-induced renin release; and compared to a candesartan treated control group. After 7 weeks of treatment *in vivo* kidney function, oxygen metabolism and mitochondrial function were evaluated. T_3_ did not affect glomerular filtration rate or renal blood flow, but increased total kidney oxygen consumption resulting in cortical hypoxia. Nephropathy, demonstrated as albuminuria and tubulointerstitial fibrosis, developed in T_3_-treated animals. Mitochondria uncoupling mediated by uncoupling protein 2 and the adenosine nucleotide transporter was demonstrated as a mechanism causing the increased kidney oxygen consumption. Importantly, blood glucose levels, mean arterial blood pressure and oxidative stress levels were not affected by T_3_. In conclusion, the present study provides further evidence for increased kidney oxygen consumption causing intrarenal tissue hypoxia, as a causal pathway for development of nephropathy.

## Introduction

Diabetes mellitus and hypertension are leading causes for development of nephropathy and subsequent end-stage renal disease (ESRD) [1]. Currently, there is no treatment to reverse already established nephropathy. Despite intense research the detailed mechanisms for development of nephropathy are unclear. However, kidney tissue hypoxia is a common finding in animal models of diabetes, hypertension and chronic kidney disease [2–7]. Thus, intrarenal hypoxia has been proposed to be a unifying pathway for development of nephropathy [8]. This hypothesis has gained increasing support [9–14] and is now referred to as “the suffocating kidney” [15]. However, when investigating the role of kidney hypoxia confounding factors such as hypertension, hyperglycemia or oxidative stress must be considered.

In most tissues, increased metabolism and oxygen consumption (QO_2_) results in increased blood flow to match oxygen supply to oxygen demand. It is therefore uncommon that increased metabolism causes hypoxia. However, the kidney constitutes an exception. Correcting increased kidney QO_2_ by increasing the renal blood flow (RBF) may result in increased glomerular filtration rate (GFR), and thus increased tubular electrolyte load. The resulting increase in active tubular transport requires increased QO_2_ *per se*, and the kidney is therefore poorly adapted to compensate for increased QO_2_ [16]. Consequently, increased kidney QO_2_ can result in decreased kidney oxygen tension [17].

Thyroid hormones stimulates increased metabolism throughout the body. Pathologically increased production of thyroid hormones, so called hyperthyroidism, causes clinical symptoms of hyperactivity of multiple organ systems such as tremor, palpitations, dyspnea, anxiety, weight loss (despite increased appetite), heat intolerance and increased sweating [18]. Thyroid stimulating hormone (TSH), secreted from the pituitary gland, stimulates the endocrine function of the thyroid to produce the thyroid hormones thyroxine (T_4_) and triiodothyronine (T_3_). T_4_ has only small effects on metabolism and is in most part converted to the more active form T_3_ within the thyroid or in peripheral target tissue. The receptors for thyroid hormones are nuclear receptors present in most cells, stimulating proliferation, differentiation and increased energy expenditure [19, 20]. Apart from direct effects on transcription thyroid hormones can also regulate activity of signaling pathways and exhibit direct effects on various organelles in the cell, including the mitochondria [20].

The constant work of reabsorption and secretion in the tubular system requires a high supply of energy, which in the proximal tubule is exclusively produced during oxidative phosphorylation by the mitochondria. The process is oxygen consuming and, therefore, changes in mitochondria metabolism have substantial effects on total kidney QO_2_. Under pathological conditions, increased mitochondria leak respiration, i.e. QO_2_ unrelated to the production of energy, has been shown to contribute to the development of kidney hypoxia [7, 21, 22].

Hyperthyroidism increases cardiac output by both inotropic and chronotropic mechanisms. An increased cardiac output together with dilation of resistance arterioles increases RBF. This increases the hydrostatic pressure in the glomerulus, thus increasing GFR and the filtered load of electrolytes to the tubular system. Further, hyperthyroidism increase proximal tubule reabsorption by stimulating the activity of several transport proteins, including the Na/K-ATPase, the Na/H-exchanger and Na-P co-transporter [23]. Hyperthyroidism also activates the renin-angiotensin-aldosterone system (RAAS). T_3_ increase renin mRNA and stimulate renin release from isolated juxtaglomerular cells [24]. Plasma renin activity, as well as serum levels of angiotensin-converting enzyme (ACE) is increased in rats [25–27] and rabbits [28] by treatment with thyroid hormones. Increased plasma renin activity has also been demonstrated in patients with hyperthyroidism [29]. Activation of RAAS promotes sodium reabsorption, increased plasma volume and vasoconstriction. Taken together, a hyperthyroid state, with increased activity of tubular transport, increased GFR and activation of RAAS substantially increases the metabolic demand of the kidney.

We have previously shown that treatment with thyroid hormones for 10 days induce proteinuria, increase total kidney QO_2_ and decrease oxygen availability in cortical tissue [30]. We hypothesized that long-term treatment with T_3_ to increase kidney metabolism in healthy rats would result in decreased kidney oxygen tension due to increased QO_2_ and ultimately lead to nephropathy. Since we have previously demonstrated increased QO_2_ by isolated kidney cortex mitochondria as a mechanism of increased total kidney QO_2_ in diabetic animals, we also studied the *in vitro* oxygen metabolism of isolated mitochondria. By using this model we aimed to investigate the role of kidney tissue hypoxia for the development of nephropathy independently of confounding factors such as hypertension, hyperglycemia and oxidative stress.

## Materials and methods

### Animals, treatment and experimental groups

All animal procedures were approved by the local animal ethics committee for Uppsala University. Animal health and well-being was assed daily in accordance with national guidelines for the care and use of animals in research. The humane endpoints was not reached for any animal during the experimental period. Adult male Sprague-Dawley rats (Charles River, Sulzfeld, Germany) weighing 290–325 grams were randomized into two treatment groups, either receiving T_3_ (10 μg/kg bw/day, osmotic minipumps, Alzet, Cupertino, CA, USA) for seven weeks in combination with the angiotensin II AT_1_-receptor antagonist candesartan (1 mg/kg in the drinking water, AstraZeneca, Mölndal, Sweden) to block the effects of T_3_-induced renin release; or candesartan only (control). Blood glucose was measured using a reagent test strip (MediSense, Bedford, MA, USA) in a blood sample obtained from the cut tip of the tail. Animals were divided into two groups for either *in vivo* kidney function measurements (n = 11 per group) or *in vitro* kidney mitochondria function measurements (n = 8 per group). All animals were housed under controlled conditions with a 12 hour light/dark cycle with free access to standard rodent chow and water.

### *In vivo* surgical procedures

Animals were anaesthetized with sodium thiobutabarbital (Inactin, 120 mg/kg bw, i.p) and placed on a servo-controlled heating pad to maintain body temperature at 37°C. Tracheotomy was performed and polyethylene catheters placed in the carotid artery and femoral vein to allow monitoring of blood pressure (Statham P23dB, Statham Laboratories, Los Angeles, CA, USA), blood sampling and infusion of saline (5 ml/kg bw/h). The left kidney was exposed by a subcostal flank incision and immobilized in a plastic cup. The left ureter and bladder were catheterized to allow for timed urine sampling and urinary drainage, respectively. Surgery was followed by a 40 min recovery period and a 50 min experimental period.

### *In vivo* measurements of kidney function and oxygen metabolism

Kidney function parameters and urine sampling were all done on the left kidney as follows. Kidney oxygen tension in the cortex and the medulla was measured using Clark-type oxygen electrodes (Unisense, Aarhus, Denmark). GFR and RBF were measured by clearance of ^3^H-inulin and ^14^C-para-aminohippuric acid (185 kBq bolus followed by 185 kBq/kg bw/h, American Radiolabelled Chemicals, St Louis, MO, USA), respectively. Liquid scintillation technique was used to determine ^3^H and ^14^C activity. Blood samples were taken from the left renal vein using a syringe and from the catheter in the carotid artery to analyze venous and arterial blood gas parameters (iSTAT, Abbott, Princeton, NJ, USA). Urinary Na^+^ and K^+^ concentrations were determined by flame photometry (IL943, Instrumentation Laboratory, Milan, Italy).

### Calculations

GFR was calculated from inulin clearance = ([inulin]_urine_*urine flow)/[inulin]_plasma_ and RBF with PAH-clearance adjusted for haematocrit and arterio-venous PAH extraction. Total kidney QO_2_ (μmol/min/kidney) was estimated from the arteriovenous difference in oxygen content (O_2_ct = ([Hb]*oxygen saturation*1.34 + pO_2_*0.003))*RBF. Tubular Na^+^ transport (TNa^+^, μmol/min/kidney) was calculated as follows: TNa^+^ = [PNa^+^]*GFR-UNa^+^V, where [PNa^+^] is plasma Na^+^ concentration and UNa^+^V is the urinary Na^+^ excretion. TNa^+^ per QO_2_ was calculated as TNa^+^/QO_2_, being a measure of sodium transport efficiency.

### Mitochondria isolation procedure

Animals were decapitated and the kidneys rapidly excised and placed in ice-cold isolation buffer (containing in mM: 250 sucrose, 10 (4-(2-hydroxyethyl)-1-piperazineethanesulfonic acid (HEPES), 1 ethylene glycol tetraacetic acid (EGTA), 1 mg/ml bovine serum albumin (BSA, further purified fraction V), pH 7.4 and osmolality adjusted to 300±2 mOsm/kg H_2_O (Model 3MO, Advanced Instruments, Norwood, MA, USA). Tissue samples were snap frozen in liquid nitrogen for analysis of reactive oxygen species and placed in Carnoy’s fixative (methanol:chloroform:acetic acid, 6:3:1) for histological evaluation. Remaining kidney cortex was separated on ice, homogenized using a cooled Potter-Elvehjelm homogeniser rotating at 600–800 rpm and centrifuged at 800xg for 10 min at 4°C. The supernatant was transferred to new tubes and centrifuged at 8000xg for 10 min at 4°C. The pellet was washed once, carefully removing the buffy coat, and then stored as a pellet on ice.

### Mitochondria respiration measurements

Mitochondria QO_2_ was evaluated in an Oroboros O2k (Oroboros Instruments, Innsbrück, Austria) in respiration buffer (containing in mM: 68 sucrose, 198 mannitol, 2 EGTA, 5 MgCl_2_, 5 KPO_4_^-^ (from a 1M mix of K_2_HPO_4_^-^ and KH_2_PO_4_^-^), 10 HEPES, 3 mg/ml BSA, pH 7.1, 330 mOsm/kg H_2_O) in the presence of 10 mM glutamate to obtain resting state QO_2_ and 300 μM ADP (pH 7.4, 0.6 mol MgCl_2_ per mol ADP) to obtain maximal QO_2_ due to oxidative phosphorylation. Mitochondria viability was assessed by the respiratory control ratio (RCR); fold increase in resting state QO_2_ in response to ADP and only mitochondria with an RCR above 4 were used in experiments. QO_2_ was evaluated in the presence of ATP-synthase inhibitor oligomycin and the effects of uncoupling protein-2 (UCP-2) inhibitor guanosine diphosphate (GDP, 0.5 mM) and adenine nucleotide transporter (ANT) inhibitor carboxyatractylate (CAT 5 μM) were investigated to estimate mitochondria leak respiration. QO_2_ was corrected for protein content in a sample obtained from the respiratory chamber at the end of each experiment.

### Urine and tissue analysis

Thiobarbituric acid reactive substances (TBARS) was measured in urine and tissue homogenate of kidney cortex by adding 50 μl sample to 42 μl 0.67% thiobarbituric acid. Samples were vortexed and heated to 97°C for 60 min. After cooling the samples on ice, 50 μl methanol:1 mM NaOH (91:9) was added, the samples vortexed and centrifuged at 3000 rpm for 5 min at room temperature. The supernatant was analyzed for fluorescence using excitation/emission of 532/553 nm and the concentration calculated using a standard curve of malondialdehyde. All values were corrected for protein concentration. Concentration of TBARS in urine samples were multiplied by urine flow to get urinary TBARS excretion. Protein content was determined by DC-Protein Assay (Bio-Rad Laboratories, Hercules, CA, USA). Albuminuria was measured by rat albumin ELISA kit, (Bethyl Laboratories, Montgomery, TX, USA) according to manufacturer´s instruction. Protein carbonylation in kidney cortex was determined spectrophotometrically by protein Carbonyl Colorimetric Assay (Cayman Chemicals, MI, USA) and normalized for protein concentration.

### Histology

Carnoy-fixed tissue was paraffin-embedded and three micrometer sections of the kidney tissue were stained with the periodic acid-Schiff (PAS) reagent and counterstained with hematoxylin.

### Statistical analysis

The data from kidney *in vivo* and mitochondrial *in vitro* measurements was assumed to be normally distributed and statistical comparisons were performed using Student’s t-test (two-tailed, equal variance). All values are given as mean±SEM. Histological sections were analysed for the presence or absence of tubulointerstitial fibrosis. Results were analysed using Fisher’s exact test (one-tailed). P<0.05 was considered statistically significant for all comparisons.

## Results

**Fig 1:**
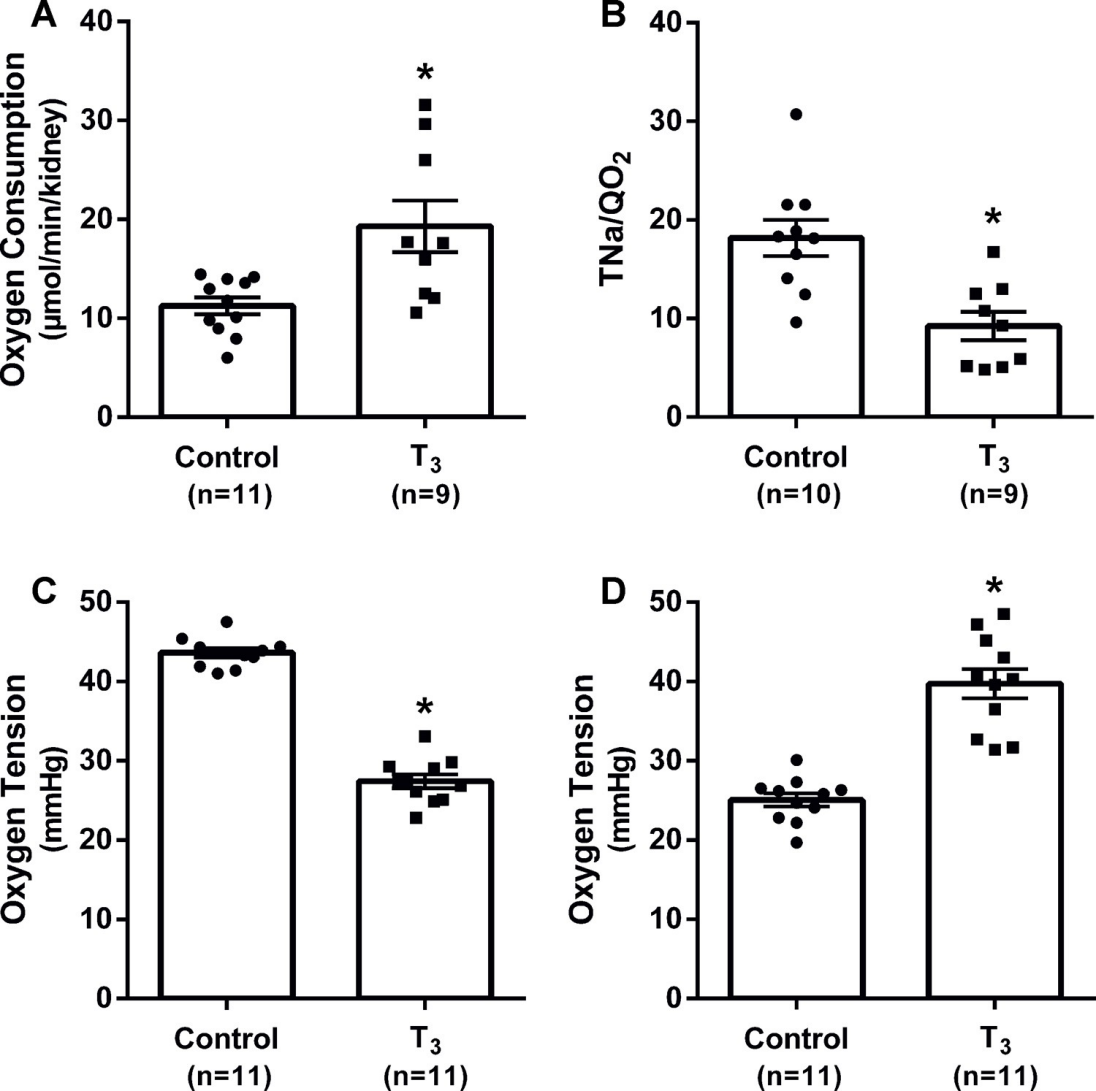
*In vivo* measurements of kidney function and oxygen metabolism in rats with and without chronic administration of triiodothyronine (T_3_). Total kidney oxygen consumption is shown in (A); transported sodium per consumed oxygen (TNa^+^/QO_2_) in (B); kidney oxygen tension in kidney cortex in (C); and medulla in (D). * denotes P<0.05 compared to control (two-tailed Student’s t-test).

Treatment with T_3_ to induce hyperthyroidism increased kidney QO_2_ and decreased the transport efficiency of sodium (TNa^+^/QO_2_) compared to control animals (Fig 1A and 1B). T_3_ decreased tissue oxygen tension in kidney cortex and increased oxygen tension in the medulla (Fig 1C and 1D). Kidney weight, GFR, RBF, urine flow and excretion of TBARS were not affected by T_3_. T_3_ decreased the filtration fraction and urinary excretion of sodium and potassium (Table 1).

**Table 1:** *In vivo* kidney function in rats with and without chronic administration of triiodothyronine (T_3_) for seven weeks.

	KW/BW (mg/g)	GFR (ml/min/kidney)	RBF (ml/min/kidney)	FF	UV (μl/min/kidney)	U-Na^+^ (μmol/min/kidney)	U-K^+^ (μmol/min/kidney)	U-TBARS (fmol/min/kidney)	C-TBARS (μmol/mg)	C-Protein carbonyls (nmol/mg)
Control	3.4±0.1	1.4±0.1	11.1±0.8	0.24±0.03	2.9±0.3	0.4±0.01	0.9±0.1	15.7±2.5	1.0±0.2	2.2±0.4
T_3_	3.2±0.2	1.2±0.1	12.7±1.6	0.16±0.02*	3.1±0.3	0.2±0.01*	0.5±0.1*	15.4±1.9	0.8±0.2	1.9±0.3

KW/BW = kidney weight per bodyweight, GFR = glomerular filtration rate, RBF = renal blood flow, FF = filtration fraction, UV = urine flow, U-Na^+^ = urinary excretion of sodium, U-K^+^ = urinary excretion of potassium and U/C-TBARS = urinary excretion and cortical tissue concentration of TBARS. All values are mean±SEM.

* denotes P<0.05 compared to control.

**Fig 2:**
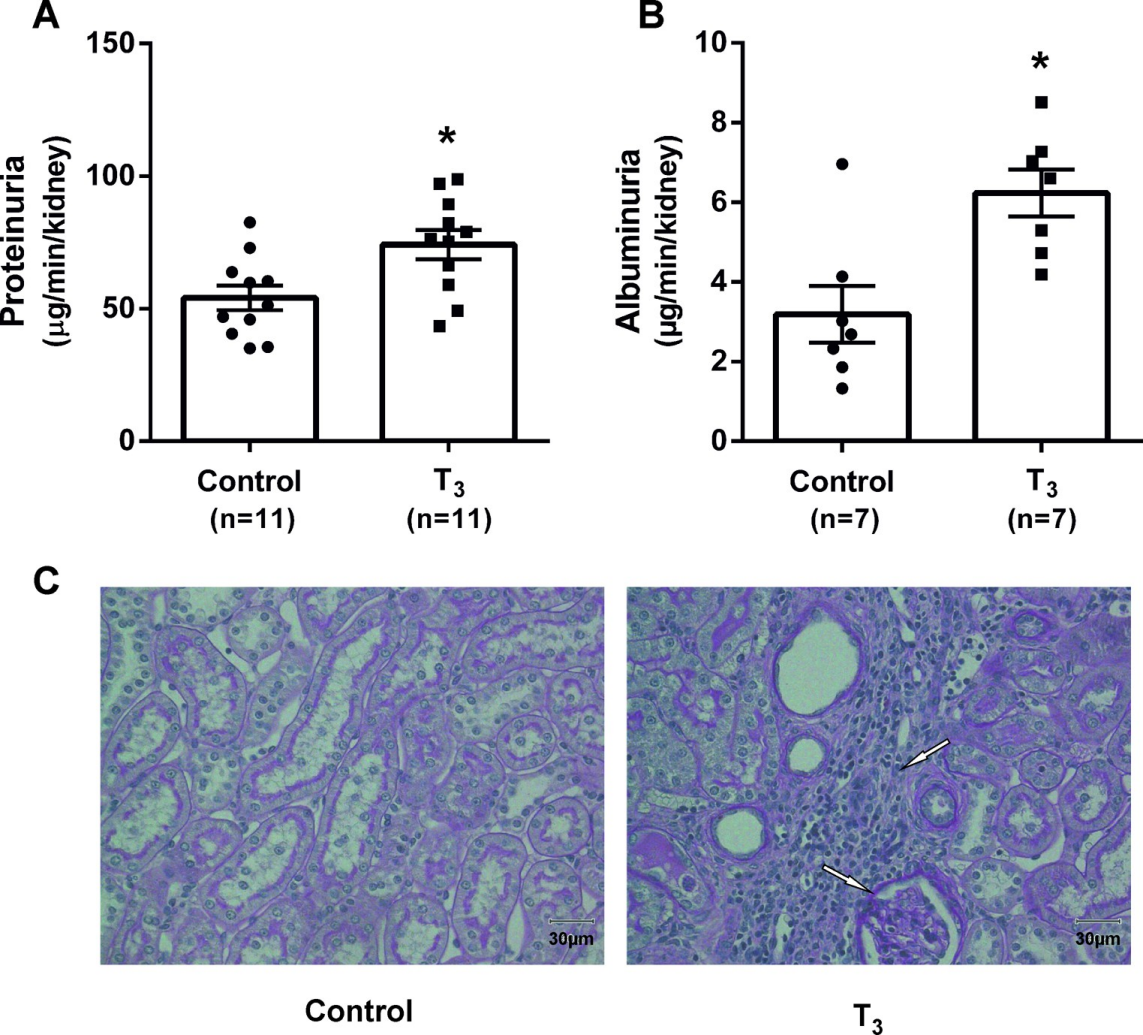
Markers of nephropathy in urine and kidney tissue from rats with and without chronic administration of triiodothyronine (T_3_). Urinary excretion of total protein is shown in (A); and urinary excretion albumin in (B). * denotes P<0.05 compared to control (Student’s t-test). A representative image of histological sections for analysis of fibrosis development are shown in (C). Focal tubulointerstitial fibrosis was present in five out of eight T_3_-treated animals whereas fibrosis could not be detected in any of the control animals (P<0.05, one-tailed Fisher’s exact test).

Excretion of protein in urine and development of tissue fibrosis was evaluated as markers of developing nephropathy. Proteinuria and albuminuria were increased by T_3_ (Fig 2A and 2B). Treatment with T_3_ induced tubulointerstitial fibrosis (Fig 2C).

**Fig 3:**
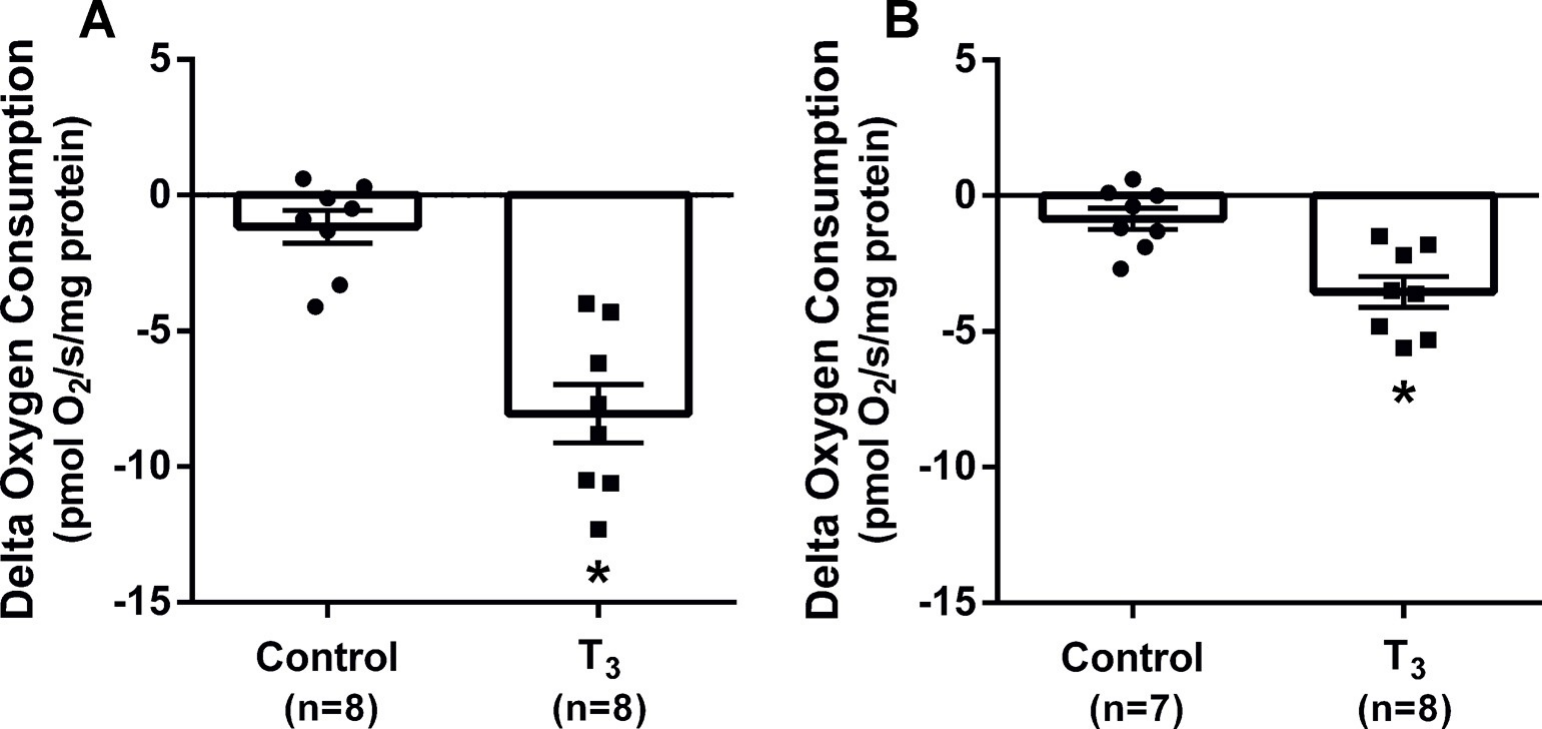
*In vitro* respiration measurements of mitochondria isolated from kidney cortex in rats with and without chronic administration of triiodothyronine (T_3_). Absolute delta change in oxygen consumption of mitochondria, after incubation with GDP to inhibit uncoupling protein 2 are shown in (A) and after incubation with CAT to inhibit adenine nucleotide translocator are shown in (B). * denotes P<0.05 compared to control (two-tailed Student’s T-test).

Kidney cortex mitochondria isolated from rats administered T_3_ had increased maximum oxidative phosphorylation capacity (respiratory control ratio, RCR) compared to controls (5.6±0.3 vs 4.6±0.2; P<0.05). QO_2_ in the presence of the ATP-synthase inhibitor oligomycin detects QO_2_ unrelated to ATP-production (leak respiration), i.e. uncoupling of the mitochondrial membrane (regulated leak) or basal leak of protons over the membrane (unregulated leak). There was a two-fold increase in absolute QO_2_ during oligomycin incubation in animals treated with T_3_ compared to controls (pmol/O_2_/min/mg protein; 29.5±2.0 vs 14.1±2.7; P<0.05), an indicator of increased total leak respiration. The change in QO_2_ after addition of the UCP inhibitor GDP and the ANT inhibitor CAT detects the degree of uncoupling via UCP-2 and ANT, respectively. T_3_ treated animals had increased uncoupling via UCP-2 (Fig 3A) and ANT (Fig 3B). The remaining QO_2_ after addition of oligomycin, GDP and CAT detects QO_2_ related to unregulated leak of protons over the mitochondrial membrane. This was increased by T3 treatment compared to controls (pmol/O_2_/min/mg protein; 17.9±2.4 vs 9.9±1.1; P<0.05), indicating an increased basal leak.

Although treatment with T_3_ induced an increased food and water intake the weight gain per week was the same between groups. Systemic parameters like blood glucose, mean arterial blood pressure (MAP), heart weight, plasma sodium and potassium were not affected by T_3_ treatment (Table 2).

**Table 2:** *In vivo* systemic parameters in rats with and without chronic administration of triiodothyronine (T_3_) for seven weeks.

	Weight gain (g/day)	Blood glucose (mM)	MAP (mmHg)	Plasma Na^+^ (mM)	Plasma K^+^ (mM)	HW/BW (mg/g)
Control	6.3±0.4	5.1±0.2	104.0±3.5	139.4±0.5	4.3±0.1	2.5±0.1
T_3_	6.4±0.3	4.8±0.2	97.4±3.7	138.9±0.4	4.1±0.1	2.7±0.1

MAP = mean arterial pressure, HW/BW = heart weight per body weight. All values are mean±SEM. * denotes P<0.05 compared to control.

## Discussion

The main finding of the present study is that increased kidney QO_2_ results in sustained kidney cortical hypoxia and development of nephropathy, manifested as albuminuria and tubulointerstitial fibrosis. Further, we show one causative mechanism being increased QO_2_ unrelated to ATP production by mitochondria. This was independent of alterations in arterial blood pressure, glycaemic status and oxidative stress. These results provide further support for kidney tissue hypoxia as an independent pathway for development of nephropathy. We have previously shown the short term effects of thyroid hormones on kidney metabolism and development of kidney hypoxia [30]. We now extend this knowledge, demonstrating that the long-term effects of increased kidney oxygen consumption and kidney tissue hypoxia leads to kidney damage.

T_3_ induce renin release and activate the renin-angiotensin-aldosterone system (RAAS), resulting in increased levels of angiotensin II signalling, hypertension and oxidative stress [31–33]. To prevent renin induced angiotensin II signalling, animals in the present study were co-treated with the angiotensin II receptor blocker candesartan throughout the study. This allowed us to study the role of kidney tissue hypoxia *per se* without the influences of confounding factors such as hypertension or oxidative stress.

Kidney QO_2_ has been demonstrated as one important factor determining intrarenal oxygen availability [17], and we have previously demonstrated a close relationship between QO_2_ and oxygen availability [7, 22, 34, 35]. The inability of the kidney to match oxygen demand to delivery originates from the close relationship between RBF and GFR, the tubular electrolyte load requiring energy-demanding tubular transport, and the structural alignment of arteries next to veins allowing for arterio-venous oxygen shunting [36, 37]. Therefore, increased kidney QO_2_ is often associated with development of kidney tissue hypoxia.

In this study, long-term treatment with T_3_ increased *in vivo* kidney QO_2_ and induced cortical hypoxia. The importance of cortical hypoxia has been demonstrated in CKD patients where decreased oxygen availability in the cortex, but not medulla, correlated with eGFR decline [38]. Also, the subgroup of CKD patients with the lowest estimated cortical oxygenation had a three times higher risk of major renal adverse events (i.e. initiation of renal replacement therapy or a 30% increase in serum creatinine).

We demonstrate that one contributing factor for the increased kidney QO_2_ is increased mitochondria uncoupling via UCP-2 and ANT. Mitochondrial uncoupling has been suggested as a protective mechanism against excessive production of superoxide radicals by decreasing the membrane potential [39–41]. However, this is done at the cost of increased QO_2_ to maintain ATP production. Thyroid hormones are known to increase heat production in brown adipose tissue by inducing uncoupling of the mitochondrial membrane via UCP-1. We now extend this knowledge to also include thyroid hormones as an inducer of UCP-2 uncoupling in the rat kidney.

UCP-2 is upregulated and activated in diabetic kidneys, where it uncouples the mitochondrial membrane and increases QO_2_ [7, 42, 43]. Furthermore, mitochondria uncoupling is also present in kidneys from hypertensive animals [44], suggesting uncoupling as a pathway to increased kidney QO_2_ also in hypertension. Oxidative stress is a known inducer of mitochondrial uncoupling [45, 46]. However, in our study oxidative stress was not increased by T_3_ treatment. The uncoupling properties of thyroid hormones could be via direct actions on the mitochondria as T_3_-receptors have been localized to the inner mitochondrial membrane [47] and it has been shown that T_3_ can directly control UCP-2 expression [48] and induce mitochondria uncoupling within minutes [49]. Thyroid hormones also bind to nuclear receptors that stimulate transcription of mitochondria specific genes [20]. Thus, a genomic effect cannot be excluded.

This study also provides evidence that T_3_ increased basal leak of protons over the mitochondrial membrane, which is another process that increases total mitochondria QO_2_. The phospholipid composition in the mitochondrial membrane determines the proton conductance over the membrane [50]. Thyroid hormones alters the composition of the mitochondrial membrane by decreasing the content of linoleic acid, a fatty acid with a strong negative correlation with basal proton leak [51]. Taken together, mitochondria uncoupling and increased basal leakage provides a mechanistic explanation for increased QO_2_ and decreased efficiency for tubular sodium reabsorption seen *in vivo* in T_3_ treated animals.

T_3_ increased mitochondria RCR, indicating increased ATP-synthesis which is a known effect of thyroid hormones on mitochondrial biogenesis [20, 52]. Since thyroid hormones increase electrolyte transport in the proximal tubule by inducing the activity of several transport proteins [23], this could indicate an increased ATP demand in the proximal tubule.

A redistribution of electrolyte reabsorption to the proximal tubule could also explain the increased oxygen availability seen in the medulla of the T_3_ treated animals. Brezis *et al*. demonstrated that direct inhibition of medullary sodium transport in the thick ascending limb increased the oxygen availability in this region [53]. This was not accompanied by an increased blood flow to the medulla. Thus, increased kidney oxygen tension in the medulla may be secondary to increased proximal sodium reabsorption. This would reduce electrolyte transport in more distal segments and lower energy demand and oxygen consumed. However in our study, medullary blood flow was not assessed and therefore hemodynamic alterations in this part of the kidney cannot be excluded, although unlikely to account for the alterations observed in kidney cortex.

Increased leakage of proteins to the urine is a well-known marker for kidney dysfunction in both humans and animal models. This can be due to damage of the filtration barrier in the glomerulus and/or damage to the tubular cells responsible for protein secretion and reabsorption. Importantly, proteinuria predicts the rate of GFR decline in patients [54]. T_3_ treatment increased total proteinuria and also more specifically albuminuria, which is commonly used as a marker of glomerular injury. Early studies of patients with autoimmune hyperthyroidism done by Weetman *et al*. revealed that approximately 30% had proteinuria despite most of them being euthyroid in response to treatment [55]. Persistent proteinuria in patients with hyperthyroidism without increased levels of circulating immune complexes have been reported by others [56, 57], indicating non-reversible kidney damage in some patients. Some studies report increased prevalence of circulating antibody-complexes in patients with hyperthyroidism that can become trapped in the glomeruli and cause glomerulonephritis with resulting nephrotic proteinuria [58–60]. However, this was not confirmed by Weetman *et al*. who neither found a high prevalence of circulating immune complexes, nor an association between immune complexes and prevalence of proteinuria [55].

It was originally hypothesized by Fine *et al*. in 1998 that kidney hypoxia could induce altered kidney function and damage [8]. Since then, the concept of kidney hypoxia as a unifying mechanism for the development of nephropathy has gained further support [9–14] based on the fact that diabetic, hypertensive and animals with chronic kidney disease all present with kidney tissue hypoxia [3, 6, 32, 61–67]. Also, type 2 diabetic patients living at high altitude have increased prevalence of nephropathy compared to a corresponding patient group living at sea level without differences in arterial blood pressure, lipid status or prevalence of retinopathy [68]. Furthermore, using a non-invasive magnetic resonance technique, it has been demonstrated that both non-diabetic chronic kidney disease patients [69] and patients with diabetic nephropathy have reduced kidney oxygen levels [69, 70]. The available literature provides support for the hypothesis that kidney hypoxia has a central role in the development of nephropathy.

The role of kidney hypoxia for the development of nephropathy has been addressed in a previous study using chronic administration of the mitochondrial uncoupler dinitrophenol (DNP). DNP administered to otherwise healthy animals for 4 weeks increased kidney QO_2_ and caused similar kidney hypoxia as in the present study. Importantly, DNP induced proteinuria, damaged tubules and infiltration of immune cells [34] but a potential nephrotoxic effect of DNP *per se* could not be excluded. However, taken together with the results from the present study where T_3_ administration induced a similar degree of increased kidney QO_2_, tissue hypoxia and nephropathy we have further support for hypoxia as an important factor for development of kidney injury.

## Conclusion

Hyperthyroidism in rats for seven weeks increased kidney QO_2_ and, consequently, induced kidney tissue hypoxia and development of nephropathy, evident as albuminuria and tubulointerstitial fibrosis. These events occurred despite unaltered glycaemic status, arterial blood pressure or oxidative stress level. The present study therefore provides further support for kidney tissue hypoxia as causal pathway to nephropathy.
